# Impact on psychiatrists in intellectual disability of Court of Protection orders for section 49 (Mental Capacity Act) reports: online survey

**DOI:** 10.1192/bjb.2022.10

**Published:** 2023-06

**Authors:** Suraj Perera, Nathalie Leyland, Jonathan Coshever

**Affiliations:** 1 Avon & Wiltshire Mental Health Partnership NHS Trust, Bath, UK; 2 Severn Deanery, UK

**Keywords:** Intellectual disability, psychiatry and law, section 49 court reports, Mental Capacity Act, writing section 49 reports

## Abstract

**Aims and method:**

To gain an understanding of court orders for reports under section 49 of the Mental Capacity Act 2005 in terms of the incidence, topics instructed, time taken, impact on working practice and well-being, and support available to psychiatrists. We used Microsoft Forms to generate an online survey. Domains within the survey included demographics, number of reports, support, clinical impact and personal well-being.

**Results:**

Of the 104 psychiatrists who responded, 65.4% had been ordered to undertake a section 49 report; 51.5% of those had been asked to provide an opinion outside their subjective expertise, 25% were somewhat or fully confident in writing reports and 85% stated that they experienced stress as a result.

**Clinical implications:**

There is a need for national and local regulation of the process of ordering reports under section 49, and for psychiatrists to be trained and supported by their employers.

Psychiatrists in England and Wales work within legal frameworks, primarily the Mental Health Act 1983, as amended in 2007, and the Mental Capacity Act 2005. They may provide expert or professional witness testimony to a variety of courts – either independently or in their role for the National Health Service (NHS). The Court of Protection was established to hear cases arising from the Mental Capacity Act involving significant decisions or resolving disputes about the property, financial affairs and personal welfare of those who are known to, or may, lack capacity to make such decisions for themselves.

## What is section 49?

Under section 49 of the Mental Capacity Act 2005 for England and Wales, the Court of Protection can order reports from NHS trusts and local authorities about a person who is the subject of proceedings, ‘P’, which ‘must deal with such matters relating to P as the court may direct'. Practice Direction 14E states (at para. 7):^1^

‘Wherever practicable, before making an application for an order requiring a report under section 49, a party to proceedings should use their best endeavours to:

- make contact with an appropriate person within the relevant local authority or NHS body so they are made aware that an application is to be made; its purpose; and the issues or questions which are hoped to be addressed within the report;
- identify a named person or by reference to their office (“the senior officer”) within the relevant local authority or NHS body who will be able to receive the court order on its behalf; and
- enquire as to the reasonableness and time scales for providing the report should the court order it.’

## Impact of section 49

The issue of instructing NHS trusts in England and Wales to supply clinical opinions on patients to the Court of Protection under section 49 of the Mental Capacity Act has been a challenge to those trusts, as they are obliged to direct their psychiatrists to write the reports. In *RS v LCC & Ors* [2015], an NHS trust formally declined to provide a report following an order under section 49, objecting on many counts, including that the consultants are not court experts and should not be expected to prepare medico-legal reports and that there would be a significant burden placed on NHS resources.^2^ All concerns were robustly rebutted by District Judge Bellamy, and since that time, the frequency of such orders has anecdotally begun to rise.

A question in 2019 by Barbara Keeley MP, then Shadow Minister for Mental Health and Social Care, to the Secretary of State for Justice about the impact of such orders on NHS resources,^3^ the suspension (reversed on appeal) of Dr Richard Pool from the medical register owing to having ‘failed to restrict his opinion to areas in which he had expert knowledge or direct experience’^4^ and the evidence submitted by the British Medical Association (BMA) to the Mental Capacity (Amendment) Bill 2019 requesting that a more manageable approach is sought, given the significant shortage of psychiatrists,^5^ all illustrate the level of concern raised formally in court and in government, and felt by colleagues following the imposition of section 49.

Consultant psychiatrists supporting people in intellectual disability and older persons services continue to find themselves in professional dilemmas where they are ordered to give an opinion on a person who may not be known to them, on an issue that may be beyond their area of professional expertise or where they struggle to meet the deadline of the court. Ordinarily, under the terms and conditions of the NHS Consultant Contract 2003 this would be classified as category B work, for which the psychiatrist could charge, but charging is not permitted under section 49.

To explore the impact of section 49 orders on clinical services, we undertook an online survey of psychiatrists in intellectual disability in England and Wales.

## Method

The survey content was developed by the authors (S.P., N.L. and J.C.), all of whom are psychiatrists in intellectual disability and aware of section 49 reports. We used Microsoft Forms (Unpaid Version, © Microsoft 2020) to generate an online survey template for distribution to participants. The survey contained a maximum of 30 questions, relating to respondents’ demographic data, number of reports completed (between January 2017 and June 2020), work intensity, support frameworks, and impact on clinical practice and personal well-being. A free-text response box was available at the end of the survey for additional comments.

The online survey was distributed to consultant and career grade psychiatrists in intellectual disability across England and Wales. An email explaining the basis of the survey and providing a hyperlink was disseminated on a regional basis, using known intermediaries (for example local training programme directors or academic programme leads). The survey was open to participants from 8 September to 9 October 2020 and a reminder email was sent to maximise participation.

Survey responses were automatically collated by the Microsoft Forms software. This information was then extracted to Microsoft Excel (© Microsoft 2020) for analysis. Free-text responses were reviewed and summarised to generate qualitative themes.

### Ethics and participation consent

This was a survey of opinion in relation to the experience of psychiatrists in intellectual disability with section 49 reports. All responses were anonymous and no patient information was collected. Therefore, no ethics approval was deemed to be necessary or was sought.

## Results

### Respondent demographics

The survey received a total of 104 responses. Sixty-eight (65.4%) respondents had been ordered to undertake a section 49 report in the period from 1 January 2017 to 30 June 2020, and a total of 166 reports were written by respondents in this period.

### Section 49 report breakdown

The number of section 49 reports completed by respondents ranged from 1 to 8, with a mean of 2.44 reports per respondent. The range of subjects requested for report varied significantly (Table 1). The most frequently requested subject was ‘care and support needs’ (59 requests, 21.5%), followed by ‘accommodation and residence’ (53 requests, 19.3%). ‘Mental health’ accounted for only 31 (11.3%) of the report subjects requested. Forty-two (61.8%) respondents were asked to write reports for an individual not on their case-load.

**Table 1:** Requested subjects for section 49 reports

	Responses^a^	
	*n*	%
Care and support needs	59	21.5%
Accommodation/residence	53	19.3%
Conducting proceedings	34	12.4%
Property/finance	33	12.0%
Mental health	31	11.3%
Contact with another person	24	8.8%
Medical treatment (non-surgical)	15	5.5%
Sexual relationships	12	4.4%
Engaging in social media	5	1.8%
Surgical procedures	3	1.1%
Other	3	1.1%
Getting married	2	0.7%

a. Respondents were able to provide more than one response to this question (68 respondents provided a total of 274 responses).

### Regarding the first three reports completed by each respondent between 1 January 2017 and 30 June 2020

The 68 respondents who had written section 49 reports were asked to give additional detail regarding the first three reports they completed (133 reports). If respondents completed fewer than three, they were asked to provide information on those they had completed.

**Fig. 1:**
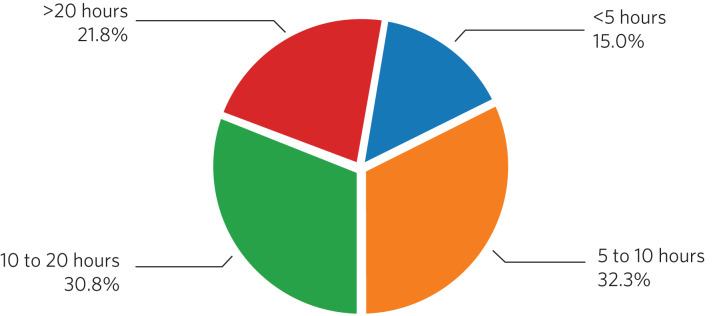
Time spent by clinicians completing section 49 reports (*n* = 133 reports).

Regarding these 133 reports, 30.8% took 10–20 h to complete and 21.8% required more than 20 h (Fig. 1). Respondents were consulted by the trust or solicitors regarding a submission date in 69 (51.9%) of the reports completed. An extension was requested for 104 (78.2%) of the 133 reports and granted in 69 (66.3%) of these cases (Table 2).

**Table 2:** Procedural framework and support for clinicians (*n* = 68 respondents) writing section 49 reports

	Responses	
	*n*	%
*Regarding all section 49 reports*		
Access to trust solicitor where necessary in completing the reports		
Yes	34	50.0%
No	16	23.5%
Don't Know	18	26.5%
Nature of support provided by trusts in completing the reports^a^		
Training	8	11.8%
Supervision	5	7.4%
No support	47	69.1%
Other	12	17.6%
Standard operating procedure in place for completing the reports		
Yes	20	29.4%
No	27	39.7%
Don't Know	21	30.9%
*Regarding up to the first 3 reports completed by respondents*		
Respondents consulted by trust solicitors regarding report submission date (*n* = 133 reports)	69	51.9%
Respondents able to request extension to submission date where necessary (*n* = 104 reports)	69	66.3%
Respondents requested to give oral evidence (*n* = 133 reports)	8	6.0%

a. Respondents were able to provide more than one response to this question (68 respondents provided a total of 72 responses).

### Procedural frameworks and support for clinicians writing section 49 reports

Thirty-four (50%) respondents who had completed reports had access to a trust solicitor and 20 (29.4%) were aware of their trust's standard operating procedure for managing section 49 reports (Table 2). The majority of respondents (47; 69.1%) stated they had ‘no support’ in providing the report (Table 2), with some stating they had ‘training’ (8; 11.8%) and/or ‘supervision’ (5; 7.4%).

‘Other’ forms of assistance were from peers, the multidisciplinary team or personal study, and may have been documented within the respondent's personal development plan (a formal agreement of the appraisee's learning needs arising from appraisal).

### Respondent expertise and confidence in section 49 report writing

When asked how confident respondents felt in preparing their first report, ‘somewhat not confident’ and ‘extremely not confident’ accounted for 37 (54.5%) of responses. We also found that 35 (51.5%) respondents were asked to provide an opinion outside their subjective expertise.

### Legal support for clinicians asked to provide oral evidence

Eleven respondents were required to provide oral evidence to the Court in support of their reports. Of these, three (27.3%) requested legal support that was declined by their trust.

### Additional outcomes

Additional outcomes from section 49 reports were reported by 14 (20.6%) respondents, including: request for an addendum; request for additional assessment (including ‘multiple’ and ‘delayed’ requests); attendance at round table meetings; and attendance at court-mandated proceedings.

### Respondent well-being and impact on routine clinical work

**Fig. 2:**
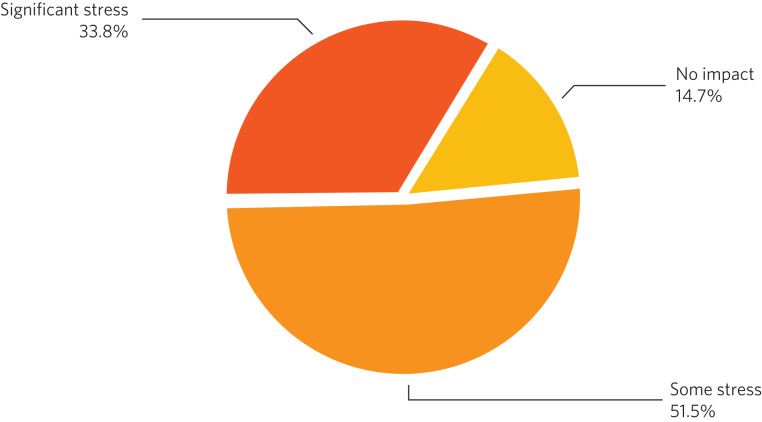
Impact of section 49 reports on mental well-being of clinicians (*n* = 68 respondents).

As regards the impact of section 49 orders on the mental well-being of clinicians, 58 (85.3%) reported ‘some’ or ‘significant’ stress. Only 10 (14.7%) respondents reported ‘no impact’ (Fig. 2).

Fifty-three (77.9%) respondents reported that there had been an impact on their routine clinical work; 15 (22.1%) reported no impact. Enquiry into the nature of this impact generated the following themes: postponement of routine reviews and non-urgent work; cancellation of clinics; use of clinical administrative time or ‘supporting professional activities’ (defined in the Consultant Contract 2003 as activities that underpin direct clinical care, such as training, medical education, research, clinical management, audit, appraisal and revalidation); increased time pressure on clinical responsibilities; use of personal time to complete reports.

### Additional respondent comments

Respondents were able to leave additional free-text comments at the close of the survey using an ‘additional comments’ option, and the main themes generated related to: regulation and administration of the process; lack of training in section 49 report writing; impact on clinician well-being; and a lack of support.

Clinicians described some requests as ‘outside’ or ‘not part of’ their ‘remit’ as a psychiatrist, resulting in reports that were ‘not an effective use’ of their time and that were ‘interfering’ with ‘NHS work’. Clinicians said it was ‘extremely difficult’ undertaking these reports on top of routine work, and that ‘no extra provision is made’ for the work section 49 reports generate. Ultimately, many clinicians described completing reports in their ‘own time’ and that this could become a ‘significant source of stress’. These issues were compounded by having ‘such tight deadlines’ and ‘no room for negotiation or extension’, with some ‘concerned’ that timescales for reports were being agreed ‘without any discussion’ with clinicians.

## Discussion

### Strengths and limitations

It is a strength that 104 psychiatrists in intellectual disability responded to the request to complete the survey, indicating the level of concern felt by colleagues. However, owing to the method of dissemination, via our own informal professional networks, we cannot be confident that the request reached all of our colleagues. Therefore we cannot provide a response rate or discuss the representativeness of the sample. The results are also subject to recollection bias, as the survey was *post hoc*, covering a period of 3.5 years.

### What the data tell us

#### The impact of a section 49 order

Over three-quarters of those who had been ordered to produce a section 49 report said there had been an impact on their work with patients, including cancellation of clinics, home visits and attendance at clinical meetings. Other essential activities also had to be postponed, such as preparation for appraisal. Many noted that they had to work on the report in their own time.

Stress was experienced by more than 75%, sometimes resulting in sick leave. Burnout has recently been reframed as end-stage ‘moral injury’, the distress experienced by clinicians when the ‘basic elements of the medical profession are eroded’, including autonomy and mastery.^6^ Autonomy over our case-load is prevented when a section 49 order is received, and mastery is threatened when the topics of the report are outside of our expertise. This is further concerning when one considers that there are already significant recruitment challenges within psychiatry of intellectual disability.^7^

#### The dilemma of being ordered to provide an opinion outside of our expertise

The most common areas that psychiatrists in intellectual disability were required to assess were capacity to consent to care and support needs, accommodation/residence, conducting proceedings and property/finance, which are more clearly within the expertise of a social worker. Those areas most relevant to the expertise of a psychiatrist, such as mental health and medical treatment, were less frequently required. We agree with the conclusion of Lindsey^8^ that greater legal weight should be placed on knowledge arising from daily professional experience and from a relationship with the person than on the traditional assumption of medical hierarchy, which priorities psychiatric evidence.

Over half of the respondents reported that they had been ordered to provide an opinion beyond their expertise, risking being in direct contravention of the General Medical Council's (GMC's) Good Medical Practice guidance:^9^ ‘You must recognise and work within the limits of your competence’ (para. 14) and ‘You must make clear the limits of your competence and knowledge when giving evidence or acting as a witness’ (para. 74).

We repeat the judgement in the above-mentioned appeal heard at the High Court following Dr Pool's 3-month suspension from the medical register because the GMC panel found that his fitness to practise was impaired: Dr Pool had provided an expert witness report outside his subspecialty and had ‘failed to restrict his opinion to areas in which he had expert knowledge or direct experience of matters that fell within the limits of his professional competence’ (*Pool v General Medical Council* [2014], para. 28).^4^

### Future work – our recommendations

#### Regulation

Regulation of the process of making orders under section 49 could be undertaken nationally and locally. We seek the support of our unions, and also the British Medical Association and the Royal College of Psychiatrists, in advocating on our behalf with the Court of Protection. We ask for national standards to be drafted, in keeping with paragraph 7 of Practice Direction 14E,^1^ requiring the Court of Protection to liaise with the proposed author of the report in order:

- to agree the issues or questions to be addressed in the report;
- to ensure that the psychiatrist is working within their expertise;
- that only the points central to the matter at hand are listed for assessment; 39 Essex Chambers has written ‘the very act of deciding to carry out a capacity assessment is not, itself, neutral, and the assessment process can, itself, often be (and be seen to be) intrusive. You must always have grounds to consider that one is necessary’;^10^ as capacity assessments are decision specific, the inclusion of each point must be justified;
- to agree the deadline for submission, usually 6 weeks from the date of the order. It should be noted that the Office of the Public Guardian requires a deadline of 6 weeks for an order of a report by a Special Visitor: an order for a report under section 49 should be treated no differently, unless there is a clinical reason for expediency.

At a local trust level, protocols covering operational and clinical procedure are required. They would include:

- managing the receipt of orders by the trust
- allocation of a clinician to undertake the assessment, including what happens when the person is not known to the trust
- negotiation of deadlines for providing the reports
- resource implications for psychiatrists in terms of time and administrative support
- recognition of the work in job planning
- access to legal support.

There should not be an expectation that this work is undertaken in the psychiatrist's own time. The fact that significant preparation may be required to ensure that all practicable steps are taken to enable the person to make the decision, that more than one appointment may be needed and that the report writing may be complex should all be taken into account.

Although our survey gained the views of psychiatrists in intellectual disability, the scope of section 49 is much wider, with our colleagues who work in older persons mental health particularly affected, and indeed any psychiatrist who works with a person who lacks capacity to make an important decision. The impact of section 49 is far ranging.

### Training

Training in report writing was a concern for many respondents to our survey. As trusts are responsible for providing the reports, it would be appropriate that they oversee training delivered by local experts, legal and social work colleagues, to include a reflective element and be undertaken on an annual basis to enable timely updates on legal precedents. Considering the proposals in reforming the Mental Health Act in England and Wales oriented towards capacity-based legislation,^11^ it is timely that psychiatrists in intellectual disability could further develop their expertise in capacity legislation.

### Concluding remarks

As psychiatrists in intellectual disability, we are committed to the welfare of our patients, who are vulnerable adults, and we wish to assist the Court of Protection in making important decisions about their health and welfare. However, the current situation is untenable – we cannot be placed in the position of being ordered to give opinions outside of our expertise, especially when there may be other professionals involved who are much better able to give such opinions. It is unreasonable that we are ordered to give such opinions within a short time frame, when the matter at hand would be considered low risk and routine, resulting in us having to step away from what may be a relatively high-risk and urgent clinical case-load. We recognise that providing reports for the Court of Protection is a specialty in itself and that colleagues require training and supervision to develop this skill.

We therefore request the support of the Court of Protection, Royal College of Psychiatrists, unions, clinicians and legal colleagues in devising a solution, which will include a national regulatory framework and training programme.

## Data Availability

The data that support the findings of this study are available from the corresponding author on reasonable request.

## About the authors

**Suraj Perera** is a consultant psychiatrist in intellectual disability with Adult Learning Disability Services, Avon & Wiltshire Mental Health NHS Trust, Bristol, UK. **Nathalie Leyland** is a consultant psychiatrist in intellectual disability with South Wiltshire Community Team for People with Learning Disabilities, Avon & Wiltshire Mental Health NHS Trust, Savernake Hospital, Marlborough, UK. **Jonathan Coshever** is a Year 6 Specialty Trainee (ST6) in the psychiatry of intellectual disability with Severn Deanery, UK.
